# Ca_v_1.3‐selective inhibitors of voltage‐gated L‐type Ca^2+^ channels: Fact or (still) fiction?

**DOI:** 10.1111/bph.16060

**Published:** 2023-03-14

**Authors:** Ludovica Filippini, Nadine J. Ortner, Teresa Kaserer, Jörg Striessnig

**Affiliations:** ^1^ Department of Pharmacology and Toxicology and Center of Molecular Biosciences University of Innsbruck Innsbruck Austria; ^2^ Department of Pharmaceutical Chemistry, Institute of Pharmacy University of Innsbruck Innsbruck Austria

**Keywords:** Ca^2+^ channel blockers, Ca_v_1.3 selective inhibitors, drug discovery, voltage‐gated Ca^2+^ channels

## Abstract

Voltage‐gated L‐type Ca^2+^‐channels (LTCCs) are the target of Ca^2+^‐channel blockers (CCBs), which are in clinical use for the evidence‐based treatment of hypertension and angina. Their cardiovascular effects are largely mediated by the Ca_v_1.2‐subtype. However, based on our current understanding of their physiological and pathophysiological roles, Ca_v_1.3 LTCCs also appear as attractive drug targets for the therapy of various diseases, including treatment‐resistant hypertension, spasticity after spinal cord injury and neuroprotection in Parkinson's disease. Since CCBs inhibit both Ca_v_1.2 and Ca_v_1.3, Ca_v_1.3‐selective inhibitors would be valuable tools to validate the therapeutic potential of Ca_v_1.3 channel inhibition in preclinical models. Despite a number of publications reporting the discovery of Ca_v_1.3‐selective blockers, their selectivity remains controversial. We conclude that at present no pharmacological tools exist that are suitable to confirm or refute a role of Ca_v_1.3 channels in cellular responses. We also suggest essential criteria for a small molecule to be considered Ca_v_1.3‐selective.

**Abbreviations**
6‐OHDA: 6‐hydroxydopamine
AP: aldosterone producing adenoma
APCC: aldosterone‐producing cell cluster
CICR: Ca^2+^‐induced Ca^2+^‐release
CPP: conditioned place preference
DA: dopamine
DHN: dorsal horn neuron
DHP: dihydropyridine
LTCC: L‐type Ca^2+^‐channel
LTP: long‐term potentiation
MLI: molecular layer interneurons
NMDAR: NMDA‐glutamate receptor
SCI: spinal cord injury
SN: Substantia nigra
VTA: ventral tegmental area

## 1 INTRODUCTION

**FIGURE 1:**
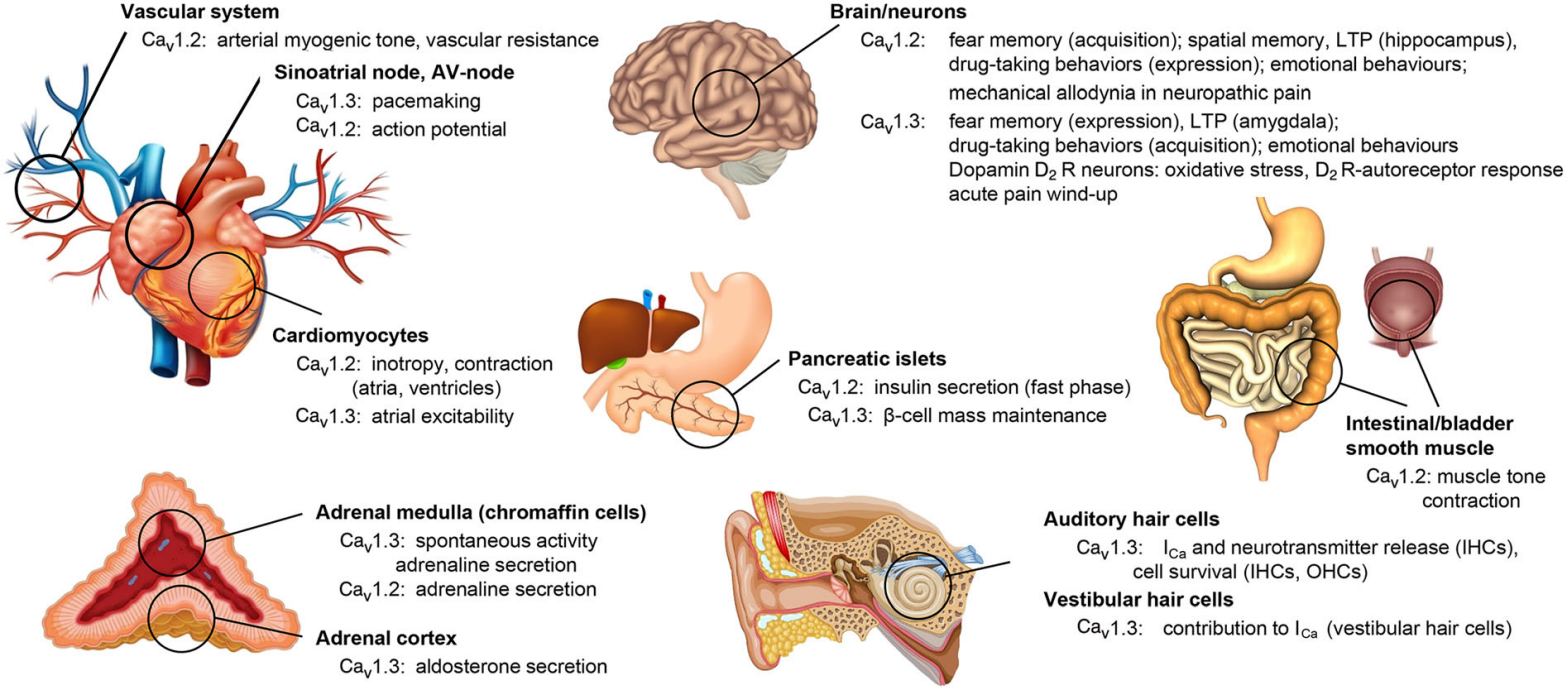
Physiological roles for Ca_v_1.2 and Ca_v_1.3 L‐type Ca^2+^‐channels (LTCCs). Ca_v_1.2 and Ca_v_1.3 are expressed in most excitable tissues and in most cases even together in the same cells. They can support different physiological functions due to their distinct gating properties (Ca_v_1.3 channels are ‘low‐voltage‐activated LTCCs’), subcellular localization and/or protein‐interactions. Ca_v_1.1 and Ca_v_1.4 show more restricted expression in skeletal muscle and retinal cells and serve key functions for skeletal muscle contraction and photoreceptor signalling, respectively (Zamponi et al., 2015). AV‐node, atrioventricular node; I_Ca_, inward Ca^2+^ current, IHC, inner hair cells; LTP, long‐term potentiation; OHC, outer hair cells. Adapted from Zamponi et al. (2015), redrawn with permission.

With the discovery of the new pharmacodynamic principle of ‘Ca^2+^ antagonism’ by substances such as verapamil and nifedipine, the German physiologist Albrecht Fleckenstein boosted research leading to the discovery of their molecular mechanism of action, the selective blockade of voltage‐gated L‐type Ca^2+^‐channels (LTCCs). LTCC inhibition in the heart and vascular smooth muscle explains their blood pressure‐lowering, anti‐anginal and antiarrhythmic actions. These clinical effects are largely mediated by Ca_v_1.2, one of four members of the LTCC family. While Ca_v_1.1 (skeletal muscle) and Ca_v_1.4 (retina) have restricted functions, Ca_v_1.3 LTCCs were found in most electrically excitable cells, and often together with Ca_v_1.2 in the same cell (Figure 1). Over the years it became clear that the biophysical properties of Ca_v_1.2 and Ca_v_1.3 as well as their relative abundance in many cells differ, which allows them to serve distinct physiological functions. The clinically relevant pharmacological effects are mediated by Ca_v_1.2 not only because of the preferential expression of Ca_v_1.2 in the cardiovascular system but also because of the high sensitivity of Ca_v_1.2 for inhibition by dihydropyridines (DHPs) in vascular smooth muscle cells due to the positive operation voltage range of these cells and the existence of splice variants favouring high sensitivity for DHPs (Liao et al., 2007; Ortner et al., 2017). Clinically available Ca^2+^‐channel blockers do not discriminate much between the two isoforms. An open questions still is whether the selective inhibition of either Ca_v_1.2 (Harrison et al., 2019) or Ca_v_1.3, which are both also widely expressed outside the cardiovascular system (including the brain and endocrine cells: Zamponi et al., 2015) has any therapeutic value. While this is unclear for Ca_v_1.2, Ca_v_1.3 appears as an attractive drug target as outlined below. Therefore, Ca_v_1.3‐selective Ca^2+^‐channel blockers are of great interest as novel tools and lead compounds for further preclinical development.

### 1.1 L‐type Ca^2+^‐channel drug binding domains

**FIGURE 2:**
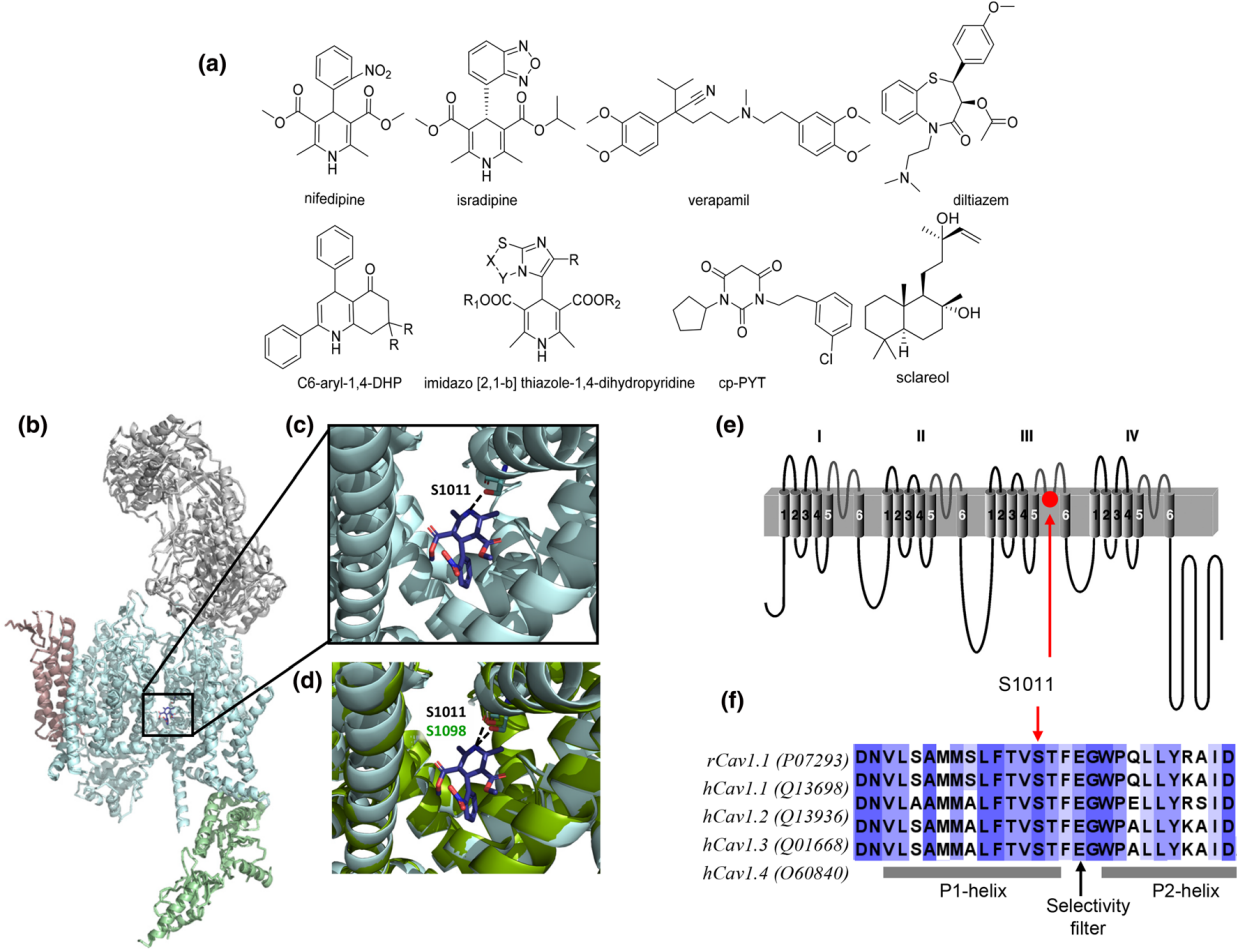
Chemical structures of compounds discussed in this article and L‐type Ca^2+^‐channel (LTCC) structure. (a) Isradipine (previously also referred to as PN200‐110) is shown as its active (S)‐(+)‐enantiomer, diltiazem as its active (+)‐cis‐diastereomer. Other names that can be found in the literature for cp‐PYT (1‐(3‐chlorophenethyl)‐3‐cyclopentylpyrimidine‐2,4,6‐(1H,3H,5H)‐trione) are compound 8 and BPN‐4689. (b) The structure of the rabbit Ca_v_1.1 channel complex with nifedipine bound in the DHP binding pocket (PDB entry 6JP5 Zhao et al., 2019). *α*1, *β*, *γ* and *α*2*δ*1 subunits are coloured pale cyan, pale green, brown and grey, respectively. The DHP blocker nifedipine is shown in dark purple. Lipids facing the DHP binding‐site has been removed for clarity. (c) DHP‐binding site in the Ca_v_1.1 structure with serine residue 1011 (numbering according to rabbit rCa_v_1.1 *α*1‐sequence, Uniprot P07293) indicated. (d) Homology model of human hCa_v_1.3 *α*1‐subunit (Uniprot Q01668) based on the rCa_v_1.1 structure, generated with MOE (Molecular Operating Environment, version 2020.09, Molecular Computing Group Inc., Montreal, Canada). The hCa_v_1.3 *α*1‐subunit (dark green) is shown in comparison to Ca_v_1.1 (pale cyan). The serine residue (corresponding to S1098 in hCa_v_1.3) represents the hydrogen‐bond acceptor partner of the hydrogen‐bond donor‐NH group in the DHP‐ring. This hydrogen bond is critical for the channel‐gating modifying activity of DHPs. (e) For orientation, the schematic transmembrane topology of Ca_v_ *α*1‐subunits is shown to highlight the approximate position of serine 1098 (red circle) in the P1 helix of repeat III in Ca_v_1.3 *α*1. F. S1011 is highly conserved in all LTCCs and located close to one of the negative charges forming the channel's selectivity filter.

All LTCCs exhibit a uniquely high sensitivity for Ca^2+^‐channel blockers. High affinity drug binding domains exist on their pore‐forming *α*1‐subunits for the three major chemical classes of Ca^2+^‐channel blockers: dihydropyridines (DHPs, e.g., nifedipine, amlodipine and isradipine), phenylalkylamines (e.g., verapamil and gallopamil) and benzothiazepines (e.g., (+)‐cis‐diltiazem) (Striessnig, 2021a; Striessnig et al., 1998) (Figure 2b–d).

The three‐dimensional structure of the Ca_v_1.1 LTCCs has recently been solved for the apo protein and in a complex with bound Ca^2+^‐channel blockers at high resolution (Gao & Yan, 2021; Wu et al., 2016; Zhao et al., 2019; for a detailed review on the structural details of voltage‐gated Ca^2+^‐ and Na^+^‐channels, see Catterall et al., 2020). An overview of the channel architecture in a complex with auxiliary subunits and with the DHP Ca^2+^‐channel blocker nifedipine is shown in Figure 2b. A serine residue in the P1 helix of repeat III forms the hydrogen‐bond interaction that is critical for DHP‐activity (Figure 2c), and it is highly conserved among all LTCCs including Ca_v_1.3. (Figure 2d–f).

**FIGURE 3:**
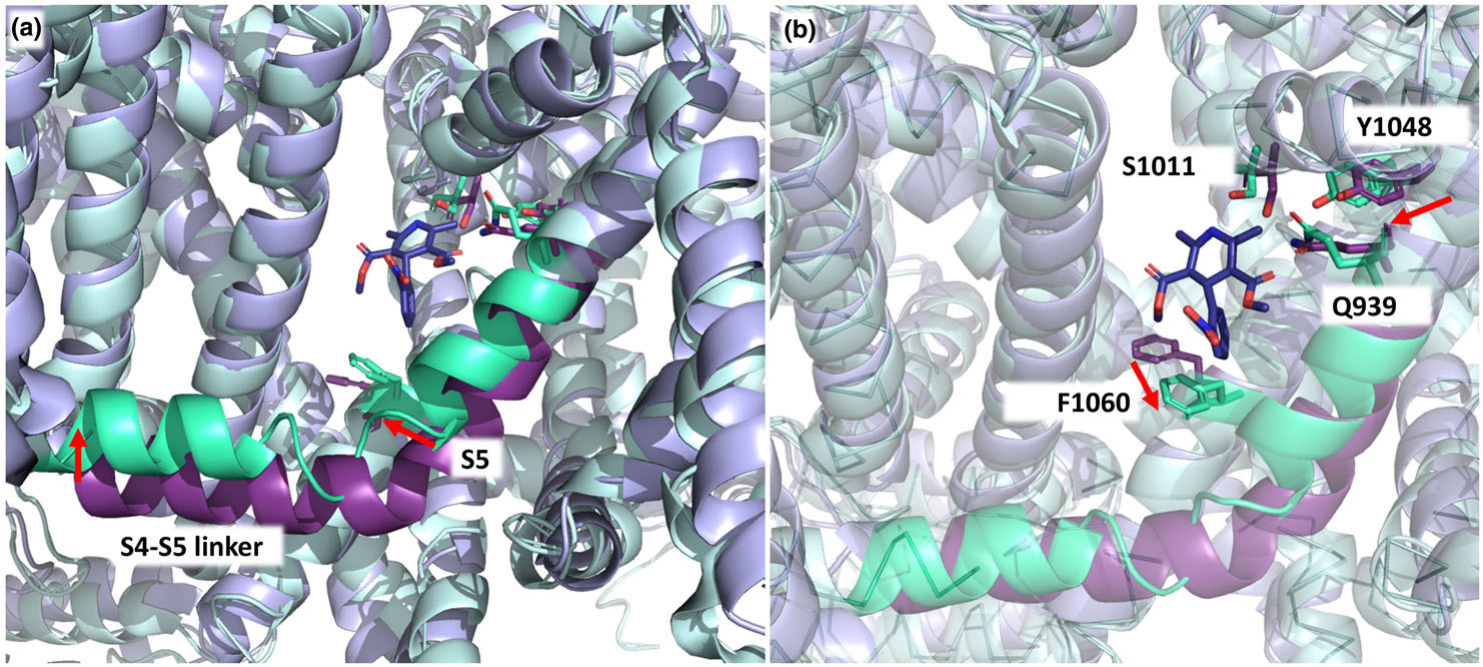
Comparison of the apo‐ and nifedipine‐bound structure of rabbit Ca_v_1.1. The apo structure is shown in violet (PDB entry 5GJV, Wu et al., 2016), whereas the nifedipine‐bound structure (PDB entry 6JP5, Zhao et al., 2019) is depicted in pale cyan. (a) In the nifedipine‐bound Ca_v_1.1 *α*1‐subunit, the IIIS4‐S5 linker is shifted upwards, whereas helix IIIS5 is rotated towards nifedipine. Red arrows indicate their movements. (b) S1011 of the repeat III pore segment, F1060 and Y1048 of IIIS6 and Q939 of IIIS5 are shown as solid sticks. They all change conformation upon ligand binding as indicated by red arrows. Please note that the S4‐S5 linker and S5 from both Ca_v_1.1 structures are coloured with a darker shade (i.e., nifedipine‐bound Ca_v_1.1 in green cyan, apo‐Ca_v_1.1 in deep purple).

Upon binding, ligands induce conformational changes within the binding site (Wu et al., 2016; Zhao et al., 2019) due to the new interactions formed with the drug (Figure 3). The S4‐S5 linker of repeat III is shifted upwards and the S5‐helix of the same repeat is rotated towards nifedipine (Figure 3a). The S6‐helix rotates as exemplified by the movement of F1060 and in addition, Y1048 moves closer to Q939 to form a hydrogen bond that is not observed in the apo structure (Figure 3b) (Gao & Yan, 2021; Zhao et al., 2019).

### 1.2 Ca_v_1.3 Ca^2+^‐channels as potential drug targets

From the four mammalian LTCC family members only Ca_v_1.2 and Ca_v_1.3 are widely expressed in essentially all electrically excitable cells (Figure 1). In contrast, expression of Ca_v_1.1 is largely restricted to skeletal muscle and Ca_v_1.4 to the retina (Zamponi et al., 2015). The Ca_v_1.2 and Ca_v_1.3 *α*1‐subunits share high sequence homology within their voltage‐sensing and pore‐forming regions including their binding domains for the different Ca^2+^‐channel blockers. Despite this close structural relationship, they differ with respect to their biophysical properties. Of important physiological relevance is the finding that Ca_v_1.3 channels activate at more negative voltages than Ca_v_1.2. Therefore, Ca_v_1.3 LTCCs underlie the ‘low‐voltage activated’ L‐type currents observed in various tissues (Avery & Johnston, 1996; Guzman et al., 2009;Mangoni et al., 2003; Marcantoni et al., 2010). Using Ca_v_1.3‐deficient mice, low‐voltage activation of Cav1.3 has been confirmed in cochlea inner hair cells, adrenal chromaffin cells, sinoatrial node cells, and pancreatic *β*‐cells (Zamponi et al., 2015). With the exception of cochlear inner hair cells, Ca_v_1.3 and Ca_v_1.2 are expressed together in the same cell but their different biophysical properties allow them to serve distinct physiological functions. For example, in chromaffin cells or sinoatrial node cells, the negative activation voltage‐range enables subthreshold Ca_v_1.3 (but not Ca_v_1.2) currents supporting spontaneous pacemaking (Mangoni et al., 2003; Marcantoni et al., 2010). Alternative splicing affects the voltage‐dependent gating of LTCCs but so far, no Ca_v_1.2 variant has been reported that activates at more negative voltages than Ca_v_1.3 variants (Ortner et al., 2017; Zamponi et al., 2015).

The pacemaking function of Ca_v_1.3 in the sinoatrial node and its absence in the ventricular myocardium predict Ca_v_1.3‐selective inhibitors as bradycardic agents lacking relevant negative inotropy (Mangoni et al., 2003; Sinnegger‐Brauns et al., 2004a) with therapeutic potential in heart failure.

Low‐voltage‐activated LTCC‐mediated Ca^2+^‐influx has also been postulated to contribute to the selective vulnerability and degeneration of substantia nigra dopamine (SN DA) neurons in Parkinson's disease (PD) (Guzman et al., 2009; Liss & Striessnig, 2019; Surmeier, Obeso, & Halliday, 2017). In SN DA neurons, each action potential triggers large dendritic Ca^2+^‐transients. In these permanently active neurons, Ca^2+^‐load enhances mitochondrial stress and thus allows LTCCs to contribute to a synergistic network of toxicity pathways and PD stressors (Guzman et al., 2009; Liss & Striessnig, 2019; Surmeier, Obeso, & Halliday, 2017). Neuroprotection with DHP Ca^2+^‐channel blockers has been observed in some, but not all, in vivo mouse PD‐models (Liss & Striessnig, 2019; Ortner, 2021). The phase‐3 STEADY‐PD clinical trial investigated the neuroprotective potential of treatment with the DHP isradipine in early PD but it did not reach its primary endpoints (Parkinson Study Group STEADY‐PD III Investigators, 2020). One likely explanation for this finding is incomplete target engagement of Ca_v_1.3 channels by the drug in the brain due to the low plasma levels reached in this study (Parkinson Study Group STEADY‐PD III Investigators, 2020) and the lower sensitivity of neuronal Ca_v_1.3‐channels for DHPs as compared to vascular Ca_v_1.2‐channels (Ortner et al., 2017). Higher dosing of isradipine or other DHPs is limited by hypotensive side effects mediated through inhibition of vascular Ca_v_1.2‐channels. Ca_v_1.3‐selective compounds may overcome this therapy‐limiting peripheral side effect.

As outlined below in this article, Ca_v_1.3 inhibition may also reduce spasticity in patients after spinal cord injury (SCI) (Marcantoni et al., 2020).

In addition, rare human disorders have provided important insight into the pathogenic role of Ca_v_1.3. Homozygous loss‐of‐function mutations in the human Ca_v_1.3 *α*1‐subunit gene (*CACNA1D*) replicate the Ca_v_1.3‐knockout phenotype observed in mice (Baig et al., 2011; Platzer et al., 2000). Affected individuals are congenitally deaf and exhibit an apparently benign sinoatrial node dysfunction. Notably, heterozygous loss‐of‐function in mice or humans is not associated with obvious disease symptoms (Baig et al., 2011; Platzer et al., 2000). In contrast, de novo (i.e., heterozygous) missense variants inducing gating changes, which also can promote Ca_v_1.3 channel activity and Ca^2+^‐influx, are pathogenic in humans (Ortner, Kaserer, et al., 2020). Such *CACNA1D* missense mutations are found as somatic mutations in a substantial fraction of aldosterone‐producing adenomas (APAs) leading to hyperaldosteronism and treatment‐resistant hypertension (Azizan et al., 2013; Korah & Scholl, 2015; Scholl et al., 2013). Interestingly, such variants are even more frequently found in small so‐called aldosterone‐producing cell clusters (APCCs) (Nishimoto et al., 2015; Omata et al., 2017). Given this key role of Ca_v_1.3‐channels in hyperaldosteronism, Ca_v_1.3‐selective inhibitors may reduce aldosterone secretion and serve as specific antihypertensives in treatment–resistant hypertension associated with APAs or APCCs (Xie et al., 2016; Yang et al., 2020).

When present in the germline, such gating‐modifying missense mutations may lead to hyperaldosteronism and/or hyperinsulinemic hypoglycemia at birth (Ca_v_1.3 channels are also expressed in pancreatic islet cells, Figure 1; Reinbothe et al., 2013; Zamponi et al., 2015). However, they are also associated with an usually severe neurodevelopmental syndrome. Symptoms include autistic behaviours, muscle hypotonia, hyperactivity, self‐aggressive behaviours, seizures and intellectual impairment (Ortner, Kaserer, et al., 2020; Striessnig, 2021b). These mutations provide unique insight into how increased Ca_v_1.3 activity can also lead to neuronal dysfunction. Selective inhibition of hyperactive Ca_v_1.3 signalling therefore appears as a potential treatment not only for the treatment of disorders with rare *CACNA1D* variants, but also for more frequent neuropsychiatric disorders, such as autism spectrum disorders or depression (Ca_v_1.3‐deficient mice display reduced depression‐like behaviours, Sinnegger‐Brauns et al., 2004a).

Both channel isoforms also play an important role in central pain sensitization, including ‘wind‐up’ as a short‐term sensitization process and allodynia in neuropathic pain. In spinal dorsal horn neurons (DHNs), LTCCs postsynaptically modulate responses to physiological or pathological stimulation (Radwani et al., 2016). Dendritic Ca_v_1.3‐channels mediate ‘wind‐up’, a progressive increase in spiking in response to nociceptive C‐fibre stimulation. They also induce plateau potentials that are followed by a sustained after‐discharge. In contrast, Ca_v_1.2‐mediated changes in gene‐transcription participate in the expression of mechanical allodynia and DHN hyperexcitability (Fossat et al., 2010). It would therefore be interesting to determine to what extent Ca_v_1.2 and/or Ca_v_1.3 inhibition could prevent the development and expression of central sensitization in neuropathic pain (Radwani et al., 2016). Ca_v_1.3‐selective inhibitors would be a valuable tool to help address this important question in preclinical pain models.

In conclusion, Ca_v_1.3‐selective Ca^2+^‐channel blockers are predicted to have a number of interesting pharmacological properties of therapeutic potential. Together these findings represent a strong rationale for discovery of potent Ca_v_1.3‐selective inhibitors.

### 1.3 Ca_v_1.3–selective Ca^2+^‐channel blockers

**FIGURE 4:**
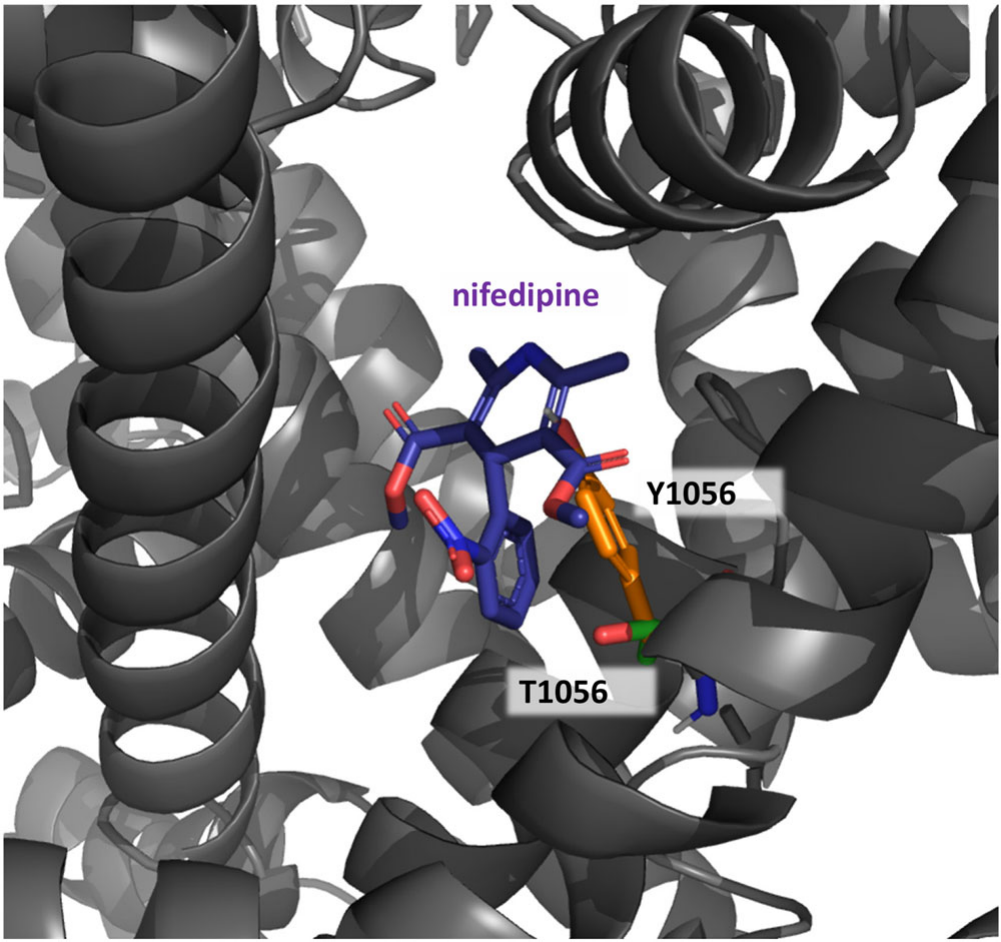
The T1056Y mutation causes a steric clash within the DHP binding site. In the model of hCa_v_1.2 harbouring mutation T1056Y (corresponding to T1066Y in mouse Ca_v_1.2; Sinnegger‐Brauns et al., 2009), the strongly reduced DHP‐sensitivity of the mutant is due to the steric clash between the Y1056 side chain and the DHP‐ring of DHPs (shown for nifedipine, blue), which prevents DHPs from accessing their binding site. This mutation has been used in in vitro and in vivo studies to prevent DHP effects through Ca_v_1.2 channels making existing DHPs ‘selective’ for Ca_v_1.3 in mutant cells. hCa_v_1.2 is shown in grey and Y1056 in IIIS5 is shown as orange sticks. Green sticks represent corresponding T1056 belonging to the wild‐type hCa_v_1.2. The homology model of hCa_v_1.2 is based on PDB entry 6JP5 (Zhao et al., 2019). Model generation and insertion of the mutant residue were conducted with MOE (see legend to Figure 2).

The pharmacology of Ca_v_1.3 Ca^2+^‐channels is complex. Among the existing drugs, DHPs are the best characterized with respect to their potential selectivity for Ca_v_1.3. Independent reports found that DHPs, such as nifedipine (Wang et al., 2018), nimodipine (Huang et al., 2013; Xu & Lipscombe, 2001) and isradipine (Koschak et al., 2001; Ortner et al., 2017) inhibit heterologously expressed Ca_v_1.3 channels with about 5–10‐fold higher IC_50_‐values than Ca_v_1.2 when studied under identical experimental protocols. For isradipine this Ca_v_1.2‐selectivity cannot easily be explained by differences in the affinity for the DHP binding pockets because in radioligand binding studies identical dissociation constants (K_D_ values) were obtained for (+)‐[^3^H]isradipine binding to recombinant Ca_v_1.3 and Ca_v_1.2 channels (Koschak et al., 2001; Sinnegger‐Brauns et al., 2009). These findings are in excellent agreement with binding studies in brain membranes from a mouse model in which high DHP‐sensitivity had been removed selectively from Ca_v_1.2‐channels by a single amino acid change (T1066Y, Sinnegger‐Brauns et al., 2004b; corresponding to T1056Y in human Ca_v_1.2‐*α*1, Figure 4). This tyrosine causes a steric clash within the DHP binding site (Figure 4) and thereby prevents high affinity interaction with DHPs. In these mice, DHPs are ‘Ca_v_1.3‐selective’ which allowed the detailed pharmacological analysis of the role of native Cav1.3 channels for many physiological functions (including behaviour, cardiac and endocrine functions) using DHPs without interference from Ca_v_1.2‐inhibition (Zamponi et al., 2015). Radioligand binding in these mice found that Ca_v_1.3‐channels comprise only about 10% of the total DHP binding sites in brain and confirmed essentially identical K_D_ values for isradipine in brain membranes as compared to heterologously expressed Ca_v_1.3 channels (Sinnegger‐Brauns et al., 2009). The differences in DHP‐sensitivity between Ca_v_1.3 and Ca_v_1.2 observed in functional studies must therefore involve conformational changes induced by changes of membrane‐voltage, protein interactions and/or alternative splicing. Ca_v_1.2 splice variants (e.g., variants predominantly expressed in cardiac vs. smooth muscle) show considerable differences in DHP‐sensitivity (Liao et al., 2007). Likewise, C‐terminal splicing in the Ca_v_1.3‐channel, which occurs outside the drug binding domains but causes profound changes in channel inactivation and voltage‐dependence of gating, strongly affects sensitivity for the DHP nimodipine (Huang et al., 2013), later also confirmed for isradipine (Ortner et al., 2017). In contrast to isradipine, some DHPs, such as nitrendipine and nifedipine, also have lower binding affinity for Ca_v_1.3, indicating subtle differences in the coordination within the two binding pockets (Sinnegger‐Brauns et al., 2009).

### 1.4 Small molecule screens in native cells

Tenti and colleagues synthesized new C5‐unsubstituted C6‐aryl‐1,4‐DHPs (Figure 2a) for which they claimed improved Ca_v_1.3 selectivity (Tenti et al., 2014). The pharmacological characterization of their compounds was based on a functional assay in native cells. Inhibition of Ca_v_1.2 was quantified through inhibition of contraction of rat mesenteric resistance arteries, that were precontracted with 70 mM K^+^. Ca_v_1.2 channels mediate contractility in arterial smooth muscle and the working myocardium (Zamponi et al., 2015), and the compound‐mediated inhibition can therefore report activity for this isoform. Inhibition of Ca_v_1.3 was quantified in fluorescent Ca^2+^ imaging assays from the inhibition of cytosolic Ca^2+^‐transients induced by depolarization with 70 mM K^+^ in undifferentiated SH‐SY5Y human neuroblastoma cells. The assumption that this assay reports Ca_v_1.3 activity, however, is not supported by sufficient evidence. Undifferentiated SH‐SY5Y cells express nifedipine‐sensitive L‐type and ω‐conotoxin GVIA‐sensitive N‐type currents (Reeve et al., 1994; Sousa et al., 2013). Both Ca_v_1.3 (Sousa et al., 2013) and Ca_v_1.2 *α*1‐subunit transcripts (Billingsley et al., 2018; Jiang et al., 2018; Sun et al., 2017; Wang et al., 2014) have been identified in these cells and their relative contribution to total L‐type current is unknown. It therefore remains unclear to what extent the inhibition of Ca_v_1.3 currents contributes to the observed inhibition of Ca^2+^‐transients, which makes it impossible to interpret isoform‐selectivity of compounds using this assay. Moreover, the action of nifedipine, and perhaps also its DHP‐derivatives, is highly voltage‐dependent (Bean, 1984; Koschak et al., 2001; Lee & Tsien, 1983). DHPs preferentially bind to inactivated channel states. Therefore, they inhibit channels with much lower IC_50_ values when a cell is held at (or fires from) more depolarized membrane potentials, which favour inactivated channel states. In this respect, assay conditions were not comparable, because their compounds were tested for Ca_v_1.2 effects in mesenteric arteries pre‐contracted (i.e., depolarized) with 70 mM K^+^, whereas they were preincubated in SH‐SY5Y cells before depolarization with 70 mM K^+^ to test for activity at Ca_v_1.3 (Tenti et al., 2014). This can also explain their finding of a 950‐fold selectivity of nifedipine for Ca_v_1.2, due to its low nanomolar IC_50_ for inhibition of mesenteric artery constriction as compared to micromolar concentrations required for half‐maximal inhibition of SH‐SY5Y Ca^2+^ transients. The very large selectivity for this Ca_v_1.2‐mediated effect was reduced to between 2‐ and 8‐fold for some of their compounds due to a much lower potency for inhibition of arterial smooth muscle constriction. This reduction in selectivity was interpreted as drastically improved ‘selectivity’ towards Ca_v_1.3 although these compounds were still, even if to a smaller extent, Ca_v_1.2‐selective. No attempts were made to test if their compounds also inhibited non‐L‐type currents in SH‐SY5Y cells nor were they tested for potency or selectivity for Ca_v_1.3 Ca^2+^‐channel currents in heterologous expression systems or in native cells, in which low‐voltage‐activated Ca_v_1.3 currents can be isolated (e.g., mouse chromaffin or sinoatrial node cells; Zamponi et al., 2015).

A similar functional screen in native cells has also been performed for a large series of DHPs with different imidazo[2,1‐*b*]thiazole substituents in position C4 (Figure 2a) (Budriesi et al., 2008; Locatelli et al., 2013). These 4‐imidazo[2,1‐*b*]thiazole‐1,4‐DHPs were screened for their negative inotropic and chronotropic effects in guinea‐pig isolated left and right atria, and for inhibition of the contraction of K^+^‐depolarized (80 mM) guinea‐pig vascular (aortic strips) and nonvascular (ileum longitudinal) smooth muscle. In contrast to nifedipine, the tested compounds were only weak inhibitors of vascular smooth‐muscle contraction. They more efficiently inhibited ileal than aortic smooth muscle but in general with higher IC_50_‐values than those required for their negative inotropic effects. Some compounds showed selectivity for negative chronotropic effects measured in right atria. In addition, binding studies were performed in membranes prepared from guinea‐pig cardiac atria and ventricles or from rat brain cortex using the (unselective) DHP Ca^2+^‐channel blocker (+)‐[^3^H]isradipine (=PN200–110) (Budriesi et al., 2008; Locatelli et al., 2013). Some compounds partially inhibited (+)‐[^3^H]isradipine binding to guinea‐pig atrial membranes with IC_50_‐values about 10–50 times lower than to guinea‐pig ventricular membranes, whereas nifedipine showed similar affinity in both tissues (Locatelli et al., 2013). However, a methodological issue was that 100 μM nifedipine displaced only 72%–80% of (+)‐[^3^H]isradipine binding although even much lower concentrations (5 μM) were used to define nonspecific binding, questioning their results from binding studies. Most of the tested compounds also partially inhibited (+)‐[^3^H]isradipine binding to membranes prepared from rat brain cortex. From these studies, the authors concluded that 1,4‐DHP scaffolds bearing a imidazo[2,1‐*b*]thiazole ring at C4 can provide compounds with cardiac selectivity less affecting vascular smooth muscle and with different actions on different heart functions, including compounds with some selectivity for negative chronotropic effects. The authors discussed different actions of these molecules on ‘heterogeneously expressed Ca_v_1.2 and Ca_v_1.3 in heart and brain’ and that ‘the substituents at the imidazo[2,1‐*b*]thiazole ring can modulate the activity of compounds on different heart functions influenced by cardiac LTCCs isoforms Ca_v_1.2 and Ca_v_1.3’. However,whether their experiments can indeed differentiate between Ca_v_1.2 and Ca_v_1.3 remains unclear. One could speculate that they consider compounds with slight selectivity for negative chronotropic effects as Ca_v_1.3‐selective because Ca_v_1.3 supports pacemaking in the sinoatrial node (Mangoni et al., 2003; Zamponi et al., 2015). However, the authors did not test their compounds in the sinoatrial node and the negative chronotropic effects may also be due to inhibition of other ion channels including T‐type Ca^2+^ channels (Torrente et al., 2020). While the experimental design provides interesting structure–activity relationship data on imidazo[2,1‐*b*]thiazole–substituted DHPs and their effect on heart, smooth‐muscle and brain function, it can provide little evidence regarding the selectivity profiles of the tested compounds in the absence of binding or functional studies with identified Ca_v_1.3 channels.

All of these studies included computational molecular modelling studies to explain the interaction of C5‐unsubstituted C6‐aryl‐ or imidazo[2,1‐*b*]thiazole – substituted DHPs (Budriesi et al., 2008; Locatelli et al., 2013; Tenti et al., 2014) with Ca_v_1.2 and Ca_v_1.3 binding pockets. However, these were based on preliminary models of the DHP binding domain constructed before the cryo‐electron microscopy structure of the Ca_v_1.1 Ca^2+^‐channel complex in its apo‐ (Wu et al., 2016) and drug‐bound‐state (Zhao et al., 2019) became available. Consequently, the authors could not take the ligand‐induced conformational changes of the channel into account (Figure 3), which are required to fit the molecules in close proximity to the highly conserved serine and allow formation of the hydrogen bond that is critical for activity (Figure 2b–f).

Taken together, the concept of defining the selectivity of compounds for Ca_v_1.2 or Ca_v_1.3 channels by performing functional and binding studies in cells or tissues without clearly defined expression and a validated isoform‐specific functional readout is of limited value and even misleading for the discovery of Ca_v_1.3‐selective inhibitors. Moreover, selectivity also needs to be confirmed in heterologous expression systems as well as in native cells with well‐characterized Ca_v_1.2 and Ca_v_1.3 current components.

### 1.5 Small molecule screens using recombinant channels

Based on the findings that LTCC inhibition by DHPs distinguishes to some extent between Ca_v_1.2 and Ca_v_1.3 (although with a preference for Ca_v_1.2), Chang and colleagues (Chang et al., 2010) synthesized a library of 124 chemically diverse 1,4‐dihydropyridines in search of structures that are potent and selective inhibitors of Ca_v_1.3. Channel inhibition was measured using tsA201 cells stably transfected with rabbit Ca_v_1.2 or rat Ca_v_1.3, either by testing the percent inhibition by a single concentration or by determining IC_50_ values in detailed concentration‐response curves using a FLIPR assay. Nifedipine served as the lead compound. Modifications included changes at the alkyl groups at the 2‐ and 6‐positions, at the 4‐position and at the amino group of the DHP ring. This should provide a detailed structure–activity relationship to identify structural features that increase potency and selectivity towards Ca_v_1.3 channels. However, despite this effort, none of the more potent inhibitors of Ca_v_1.3 exhibited relevant selectivity (<1.7‐fold). The two most selective analogues were only 2.2‐ and 2.4‐fold selective and both had only micromolar potencies.

In a follow‐up study (Kang et al., 2013a), the same authors explored diverse alternative scaffolds, including 1,4‐dihydropyrimidines and 4H‐pyrans, to introduce further structural variations. A library of 36 DHP‐mimetics was prepared, all with a 3‐nitrophenyl as the aromatic substituent (like in nitrendipine), but with modifications to the DHP‐scaffold (pyrimidinone, pyrimidinethione, and hydropyran), to each of the esters and to the alkyl side chain. The IC_50_ values for these compounds were determined by concentration–response curves in a FLIPR assay. Again, only modest improvements in selectivity could be achieved. The pure R‐enantiomer of the most potent compound inhibited Ca_v_1.3 with an IC_50_ of 0.51 μM and Cav1.2 with an IC_50_ of 1.02 μM, corresponding to a difference of only about 2‐fold.

Despite the failure to discover Ca_v_1.3‐selective lead compounds, these two studies (Chang et al., 2010; Kang et al., 2013) were very important, because they provided substantial evidence that relevant Ca_v_1.3‐selectivity cannot be achieved by modifying the substitutions of the DHP scaffold.

Therefore, Kang et al. (2012) set out to discover novel scaffolds suitable for Ca_v_1.3‐selective channel inhibition. In their FLIPR‐based high‐throughput screen they identified pyrimidine‐2,4,6‐triones, a class of compounds previously characterized as potential neuroprotective agents in amyotrophic lateral sclerosis and with favourable pharmacokinetic properties (Xia et al., 2011). Chemical modifications finally led to a Ca_v_1.3‐selective compound, termed compound 8 (1‐(3‐chlorophenethyl)‐3‐cyclopentylpyrimidine‐2,4,6‐(1H,3H,5H)‐trione) (Figure 2a). In later publications it was also termed cp‐PYT or BPN‐4689. We will use cp‐PYT to refer to this compound throughout this article. In FLIPR‐assays it inhibited Ca_v_1.3 with IC_50_‐values of 1.7–6.3 μM (Kang et al., 2012; Kang et al., 2013b) but it appeared as a weak inhibitor of Ca_v_1.2 with an estimated 612‐fold selectivity for Ca_v_1.3 (Kang et al., 2012). This was further confirmed in whole‐cell patch‐clamp experiments in HEK‐293 cells stably expressing Ca_v_1.3 or Ca_v_1.2. Cp‐PYT inhibited Ca_v_1.3 with an IC_50_ of 24.3 μM and >100‐fold selectivity over Ca_v_1.2 (Kang et al., 2012).

Unfortunately, these findings could not be reproduced by two other groups. Huang et al. (2014) studied the potency and selectivity of cp‐PYT using whole‐cell patch‐clamp recordings in transiently transfected HEK‐293 cells expressing C‐terminal long (Ca_v_1.3_42_) and short (Ca_v_1.3_42a_) rat Ca_v_1.3 *α*1‐subunit splice variants or rat Ca_v_1.2B15 (Tang et al., 2007) together with rat *α*2*δ* and the rat *β*‐subunit isoforms *β*1‐*β*4. Although recording conditions were similar (test pulses from −70 mV to 10 mV at 0.05 Hz) current inhibition by 50 μM of the compound was less than 50% for both Ca_v_1.3‐splice variants and similar to Ca_v_1.2 when *β*1‐, *β*3‐ and *β*4‐subunits formed part of the channel complex. However, upon co‐expression of *β*2a‐subunits (a palmitoylated *β*‐subunit that slows channel inactivation; Buraei & Yang, 2010; Ortner, Pinggera, et al., 2020 for review), cp‐PYT was even slightly Ca_v_1.2‐selective (Huang et al., 2014).

In addition, Ortner et al. (2014) could not observe Ca_v_1.3 selectivity using whole‐cell patch‐clamp recordings with a pulse‐protocol and assay conditions similar to those in the original paper. TsA‐201 cells were transfected with the long splice variants of rat (rCa_v_1.3L) or human Ca_v_1.3 (hCa_v_1.3L), rabbit rbCa_v_1.2 (same construct as in the original study) or rbCa_v_1.2S (truncated at amino acid position 1800 to account for proteolytically processed forms in heart and brain) together with rat *β*3 and rabbit *α*2*δ*1. In contrast to the other studies, 50 μM cp‐PYT strongly affected Ca_v_1.3 and Ca_v_1.2 current kinetics characterized by a slowing of the activation and inactivation time course as well as a pronounced slowing of deactivation upon repolarization. These changes resembled the kinetic changes induced by the LTCC activator FPL64176 (Kunze & Rampe, 1992). These effects on gating were absent only in a small minority of cells, in which cp‐PYT, however, blocked Ca_v_1.3 and Ca_v_1.2 weakly but to a similar extent. The absence of selective inhibition was not an artefact of heterologous expression because the slowing of tail current was further confirmed in native Ca_v_1.2 and Ca_v_1.3 LTCC current components in mouse chromaffin cells (Ortner et al., 2014). In summary, these results suggest, that cp‐PYT can behave as an activator rather than a blocking agent of LTCC currents. No evidence for selectivity was found for this compound.

**FIGURE 5:**
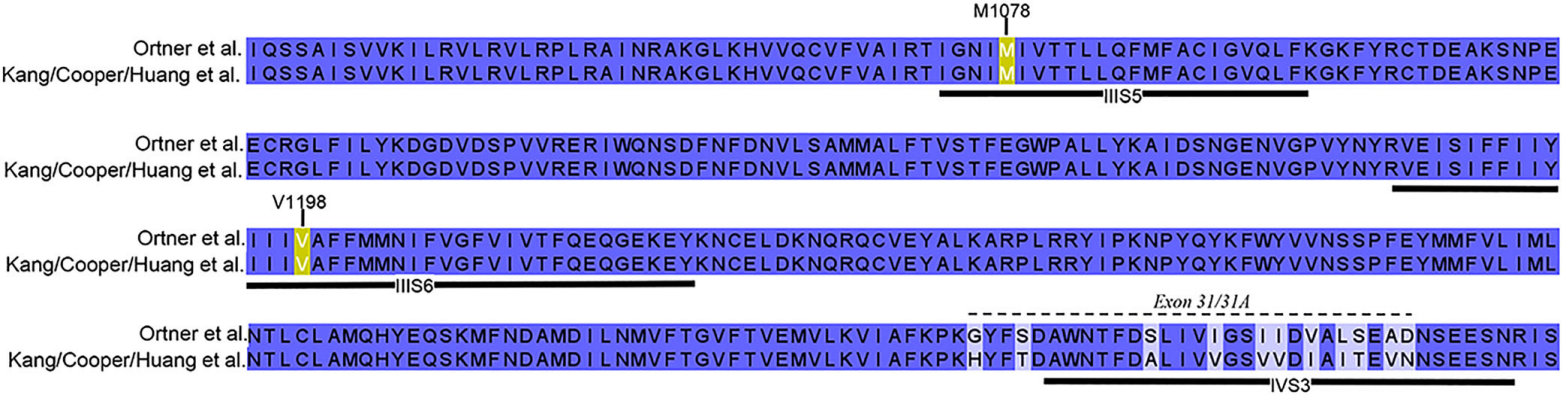
Sequence alignment of rat Ca_v_1.3 *α*1 subunit constructs used by different groups to study the selectivity of cp‐PYT. Alignments are shown for regions in homologous repeats III and IV with amino acid differences between the constructs. Note that residues M1078 and V1198 (orange) are not different (for details see text). The only difference is alternative splicing of exon 31 (Lieb et al., 2012), which was different in the construct used in the study by Ortner (Ortner et al., 2014) compared to those by the other laboratories (GenBank accession #: AF370009/AF370010; Cooper et al., 2020; Huang et al., 2014; Kang et al., 2012).

In a more recent study (Cooper et al., 2020), the authors of the original findings tried to resolve these discrepancies by studying the molecular mechanism of cp‐PYT interaction within its binding pocket on Ca_v_1.3‐channels in more detail. They confirmed the selectivity with an about 60% and 20% inhibition of Ca_v_1.3 and Ca_v_1.2 by 100 μM of the compound, respectively (again determined with whole‐cell patch‐clamp recordings as in the original study, Kang et al., 2012). A key observation was that a single amino acid exchange in Ca_v_1.3 *α*1, T1081Y (which corresponds to T1066Y in ref. Sinnegger‐Brauns et al., 2004a, see Figure 4), which is known to eliminate high affinity for DHPs (see above), also prevented cp‐PYT inhibition (100 μM). This strongly suggested that cp‐PYT and DHPs bind to the same region. This was further supported by the observation that the binding of the tritiated cp‐PYT derivative [^3^H]‐D11 to recombinant Ca_v_1.3 channels was partially displaced by the DHP isradipine (Cooper et al., 2020). The authors then constructed a homology model of Ca_v_1.2 and Ca_v_1.3 based on a cryo‐EM structure of Ca_v_1.1 (skeletal muscle) Ca^2+^‐channels (Wu et al., 2016). From their model they predicted that the selectivity for Ca_v_1.3 results from two residues within the DHP/cp‐PYT binding pocket that differ between Ca_v_1.3 and Ca_v_1.2 *α*1‐subunits (for sequence alignment see Figure 5): M1078 (in IIIS5), which forms the bottom of the binding pocket, and V1198 (located in S6 of domain III and not IVS6, as erroneously stated by the authors). Mutation of M1078 to valine, the corresponding amino acid in Ca_v_1.2, reduced the sensitivity to cp‐PYT. Conversely, replacement of V1063 in Ca_v_1.2, which corresponds to M1078 in Ca_v_1.3, by methionine increased the sensitivity of Ca_v_1.2 for the compound (about 50% inhibition by 100 μM cp‐PYT). Notably, mutation M1078V also strongly shifted the more negative current–voltage relationship of Ca_v_1.3 to more positive voltages, indistinguishable from Ca_v_1.2. Therefore, assuming state‐dependent effects of cp‐PYT (see below), the altered gating of this construct, rather than changes in its binding domain itself, may also account, at least in part, for its reduced sensitivity to cp‐PYT. Change of Ca_v_1.3 V1198 to the corresponding isoleucine in Ca_v_1.2 had no effect.

None of these mutations affected isradipine sensitivity, which is in contrast to an earlier report (Wang et al., 2018) demonstrating that M1078V in Ca_v_1.3‐*α*1 (M1030V in Wang et al., 2018) increased, whereas the reciprocal Ca_v_1.2 mutation decreased sensitivity for nifedipine.

The homology model generated by the authors (Cooper et al., 2020) provided a rational basis to predict which residues could account for the proposed selectivity of cp‐PYT towards Ca_v_1.3 and was also used to predict a potential cp‐PYT binding mode. However, the conclusions derived thereof have to be interpreted with caution since the model was based on the apo‐structure of Ca_v_1.1 (Wu et al., 2016) rather than on the structure of the drug‐bound conformation, which was already published at that time (Zhao et al., 2019), as outlined above (Figure 3). Therefore, their model predicted an isradipine‐docking pose unable to recapitulate the pose resolved for nifedipine in the cryo‐EM structure (Gao & Yan, 2021; Zhao et al., 2019).

As also shown for DHPs, cp‐PYT (10 μM) inhibition was voltage‐dependent and blocked Ca_v_1.3 more efficiently (26% versus 46% inhibition) when cells were held at more depolarized voltages (−50 versus −80 mV, Cooper et al., 2020). In acutely isolated SN DA neurons, which express Ca_v_1.3, Ca_v_1.2, Ca_v_2.3 and other voltage‐gated Ca^2+^ channels (Benkert et al., 2019; Poetschke et al., 2015), 50 μM cp‐PYT inhibited about 50% of the voltage‐gated Ca^2+^ current. Interestingly, the same concentration of cp‐PYT did not inhibit current in neurons prepared from Ca_v_1.3‐deficient mice, interpreted as evidence for Ca_v_1.3‐selectivity of the compound. However, the contribution of isradipine‐sensitive L‐type current to the overall Ca^2+^‐channel current in their SN DA neurons was not studied. Therefore, it remains unclear if a Ca_v_1.2‐mediated L‐type current component even exists in these cells that, if not inhibited by cp‐PYT, would allow to prove its selectivity.

Application of cp‐PYT (50 μM) to ex vivo midbrain slices had no effect on pacemaking of SN DA neurons, but significantly reduced the dendritic Ca^2+^ oscillations, consistent with inhibition of LTCC activity. Similar to 1 μM isradipine, cp‐PYT (50 μM) also diminished mitochondrial oxidant stress in these neurons when compared to previously published historical controls (Figure 1h in Guzman et al., 2010).

Unlike observations by others in a previous publication (Ortner et al., 2014), only very minor gating changes were found by Cooper and colleagues (Cooper et al., 2020). A very small and non‐significant (at n = 3) slowing of deactivation (less than 2‐fold increase of the deactivation time constant) was evident at higher (50 μM) but not at lower (10 μM) concentrations of cp‐PYT. This was explained by the existence of an additional low affinity site on the channel, which could mediate slowing of deactivation but not channel inhibition (Cooper et al., 2020). Indeed, from their binding data using [^3^H]‐D11, a close structural analogue of cp‐PYT, the existence of a low affinity cp‐PYT binding site cannot be excluded. This is based on the observation that isradipine only displaced about 80% of specific binding. However, it is unclear if this partial inhibition resulted from partial displacement due to overlapping or negatively allosterically coupled binding sites for [^3^H]‐D11 and isradipine, or from occupation of an additional low affinity/high capacity site by [^3^H]‐D11 unrelated to LTCCs. The data do not allow conclusions about the overall binding capacity of [^3^H]‐D11 in their membrane preparation (the Scatchard plot revealed a B_max_ of 0). Nevertheless, from their experiments the authors concluded that under their experimental conditions cp‐PYT is Ca_v_1.3‐selective and that it shares a common binding pocket with DHPs.

At the moment, it is not clear why three independent laboratories obtained different results despite testing cp‐PYT under similar experimental recording conditions. It would therefore be important to elucidate the experimental and/or molecular mechanisms to evaluate under which conditions, cp‐PYT could behave as a Ca_v_1.3 selective blocker. The use of different splice variants of the Ca_v_1.3 and Ca_v_1.2 *α*1‐subunits may contribute to the different observations. However, to date no experimental evidence has been reported to support this notion. In contrast to Cooper et al. (2020) and Huang et al. (2014), Ortner et al. (2014) employed a rat Ca_v_1.3 *α*1‐subunit construct that was alternatively spliced to contain exon 31A instead of exon 31 (Figure 5 for sequence alignment) but, unlike stated in the paper (see published correction, Cooper et al., 2021) was otherwise identical to the other rat clones. Whether alternative splicing can account for the ‘agonistic’ effects of the compound has so far not been investigated. It should be kept in mind also for future studies that selectivity ratios are also affected by the nature of the Ca_v_1.2 construct used as a reference. However, differences in the Ca_v_1.2 constructs also appear unlikely in this case as Cooper et al. and Ortner et al. used the same rabbit construct (Uniprot accession #P15381). This corresponds to the cardiac splice variant of Ca_v_1.2 (Mikami et al., 1989). It is important to note that the vascular smooth muscle splice variants of Ca_v_1.2 inactivate at more negative voltages and, depending on resting membrane potential, are 6 to 10 times more sensitive to DHPs (Liao et al., 2007) than the cardiac splice variant used in these studies (Cooper et al., 2020; Kang et al., 2012). Given the fact that cp‐PYT is believed to bind within the DHP binding pocket and to also act in a voltage‐dependent manner no clear conclusions can be drawn regarding selectivity from experiments with the cardiac splice variant alone. Huang et al. employed a Ca_v_1.2 splice variant preferentially expressed in aortic smooth muscle. It is possible (but not yet proven) that the different splicing also provided more robust block of Ca_v_1.2 by cp‐PYT in their study, which could explain the lack of selectivity.

A recent cryo‐EM study tried to reveal the molecular basis of cp‐PYT binding to Ca_v_1.3. While the structure of a cinnarizine‐Ca_v_1.3 *α*1‐subunit complex could be solved, no electron density could be observed for cp‐PYT in the cryo‐EM structure of Ca_v_1.3 incubated with 150 μM of cp‐PYT (Yao et al., 2022). The reason for this observation is unclear but may be due to the low affinity of this compound for the channel.

Given the current uncertainty regarding the Ca^2+^‐channel selectivity profile of cp‐PYT, the lack of reproducibility between different laboratories, and the absence of information on its action on other ion channel targets, we do not support its use or promote it as a Ca_v_1.3‐selective Ca^2+^‐channel blocker, in contrast to other recommendations (Roca‐Lapirot et al., 2018).

### 1.6 Use of cp‐PYT as a Ca_v_1.3‐selective tool in other studies

Until these discrepancies are resolved the evidence for cp‐PYT being a useful Ca_v_1.3‐selective compound for further studies is limited and controversial. Data in native tissues are also not conclusive. Cp‐PYT modified gating similar to LTCC‐activators in mouse chromaffin cells (Ortner et al., 2014) but showed inhibitory effects in SN DA neurons (Cooper et al., 2020). As outlined above, it is unclear if Ca_v_1.2 L‐type currents exist in these cells, therefore no conclusions about selectivity can be drawn from these experiments.

Despite these uncertainties, and the fact that cp‐PYT has not yet been investigated for its pharmacological effects on Ca_v_2 and Ca_v_3 (T‐type) Ca^2+^ channels, it has been used as a ‘Ca_v_1.3‐selective’ tool in several studies. This is not surprising, given that the scientific community is eagerly awaiting such a compound to study the role of Ca_v_1.3 as a drug target for a number of indications as outlined above.

Xie et al. (2016) used cp‐PYT to investigate the role of Ca_v_1.3 on steroidogenesis in the human adrenocortical cell line, H295R, and in primary human adrenal cells. They transfected H295R cells with wild‐type or gain‐of‐function Ca_v_1.3 channel mutants previously identified as disease‐causing in APAs (Azizan et al., 2013). These mutant channels promote higher aldosterone production compared to wild‐type‐transfected cells, which was inhibited only by very high concentrations (100 μM) of cp‐PYT. Interestingly, low concentrations of the compound almost doubled aldosterone secretion in wild‐type transfected H295R cells and also stimulated secretion in the mutants. This effect was not observed with nifedipine (Xie et al., 2016). These findings strongly suggest that at these lower concentrations cp‐PYT activated Ca^2+^‐current through Ca_v_1.3, in agreement with the channel‐activating properties in one of the studies in tsA‐201 cells (Ortner et al., 2014). As mentioned by the authors, T‐type channels also control aldosterone secretion. Based on the unknown activity of the compound on T‐type channels, its usage is not suitable to unambiguously prove a role of Ca_v_1.3 on steroidogenesis.

Ca_v_1.3 has also been implicated as a therapeutic target for SCI. After SCI most individuals develop involuntary muscle contractions, including spasms, as a chronic complication (Jiang et al., 2021; Marcantoni et al., 2020). Altered excitatory and inhibitory synaptic input into motoneurons as well as hyperexcitabilty of motoneurons seem to underlie this phenomenon. In motoneurons LTCC activity can generate persistent inward currents supporting plateau potentials from which neurons can sustain firing. This enhanced activity of plateau potentials after SCI correlates with spasticity (Marcantoni et al., 2020). Due to their more negative activation‐range and higher activity at subthreshold potentials, Ca_v_1.3 channels have been implicated in this process (Li & Bennett, 2003). This has recently been confirmed in an elegant study by showing that prolonged treatment of mice with the Ca^2+^‐channel blocker nimodipine, starting early after SCI, can prevent the development of increased muscle tone and spontaneous spasms. This is mainly due to the inhibition of Ca_v_1.3 as demonstrated by a dramatic decrease of SCI‐induced aberrant muscle activity by neuron‐specific and constitutive Ca_v_1.3‐knockout in mice (Marcantoni et al., 2020).

The involvement of LTCCs in SCI‐induced hyperexcitability was further explored in a mouse model of acute and chronic SCI (SCI) (Jiang et al., 2021). Cp‐PYT was employed in an attempt to demonstrate the specific involvement of Ca_v_1.3. At high micromolar concentrations this compound inhibited in particular long‐lasting root reflexes and plateau potentials and reduced motoneuron firings evoked by intracellular current injection in a concentration‐dependent manner. While their data suggest that cp‐PYT (like in recombinant systems) can inhibit Ca_v_1.3 channels at high concentrations, these experiments do not provide evidence for selectivity of this compound because the contribution of other L‐type currents (presumably Ca_v_1.2) in motoneurons is small (Marcantoni et al., 2020). The authors misleadingly state that low selectivity of cp‐PYT reported in the study of Huang et al. (Huang et al., 2014) ‘is due to the existence of splice variants of Ca_v_1.3 channels, which have various biophysical and pharmacological properties’. While the latter is true (see above), none of the references cited by the authors has ever investigated the effect of alternative splicing on cp‐PYT action. They also showed a strong inhibition of long‐lasting root reflexes by inhibiting NMDA‐receptors (NMDAR) with ketamine. Since no information about the activity of cp‐PYT on glutamate receptors is provided, a contribution through this mechanism cannot be excluded. In contrast to Ca_v_1.3‐knockout (Marcantoni et al., 2020), the unknown selectivity profile of this compound does not allow conclusions about which LTCC is involved in spinal spasticity after SCI.

Degoulet et al. (2016) employed isradipine and cp‐PYT to study the role of LTCCs for ventral tegmental area (VTA) synaptic plasticity and for cocaine‐associated contextual memory. Their study built on previous observations that LTCC activation during postsynaptic depolarization can induce synaptic plasticity in multiple brain areas, including the cerebral cortex, hippocampus, amygdala and striatum. In addition, systemic administration of LTCC antagonists blocks the acquisition of drug‐induced conditioned place preference (CPP), a form of Pavlovian contextual cue learning dependent on NMDAR‐mediated signalling in the VTA (Degoulet et al., 2016). Using an elegant combination of ex vivo VTA slice‐recordings and behavioural studies in rats they found strong effects of LTCC‐inhibition on synaptic plasticity in the VTA. Isradipine blocked the induction of NMDAR mediated long‐term potentiation (LTP) but also facilitated the reversal of previously induced LTP in the VTA. In behavioural experiments isradipine injected into the VTA suppressed the acquisition of cocaine‐paired contextual cue memory assessed using a CPP‐paradigm and it abolished previously acquired cocaine and alcohol CPP. Like isradipine, cp‐PYT (20 μM) also mediated depotentiation of LTP in slice recordings and abolished previously acquired cocaine and alcohol CPP. While this elegant study demonstrates a novel role for LTCCs in the VTA and provides compelling evidence for a link between the observed changes in synaptic plasticity and the behavioural outcomes, the data cannot provide evidence for a selectivity of cp‐PYT. Even if Ca_v_1.3 channels alone are involved in the observed drug effects and are inhibited by cp‐PYT, no implications for its selectivity can be made unless it is known to what extent cp‐PYT can spare Cav1.2‐mediated currents, if present at all.

In an elegant paper, Plotkin et al. (2013) employed two‐photon laser scanning microscopy and patch‐clamp electrophysiology in striatal ex vivo brain slices to study the regulation of dendritic Ca^2+^ release in striatal spiny projection neurons (SPNs), which underlies the induction of corticostriatal long‐term depression in these neurons. They found that theta‐burst‐stimulated back‐propagating action‐potentials induce a Ca^2+^‐transient in dendritic spines, which required opening of voltage‐gated Ca^2+^ channels and activation of ryanodine receptors and was thus attributed to Ca^2+^‐induced Ca^2+^‐release (CICR). Interestingly, activation of group I metabotropic glutamate receptor (mGluRs) by itself did not affect intraspine Ca^2+^‐concentration in the absence of somatic action potentials but increased the activity‐dependent intraspine Ca^2+^‐transient in proximal and distal dendrites of indirect pathway SPNs. This modulation was dependent on the activation of Ca_v_1.3 LTCCs because it could be blocked by isradipine and was absent in neurons from Ca_v_1.3 knockout‐mice (Plotkin et al., 2013). To test if CICR is dependent on Ca_v_1.3 Ca^2+^‐channels also in the absence of mGluR activation they employed cp‐PYT at a concentration of 20 μM. Only a minimal inhibition was observed and attributed to the presence of other Ca^2+^‐channels, including Ca_v_1.2 and Ca_v_2.3 also expressed in these cells (Plotkin et al., 2013). However, the data with cp‐PYT are limited by the low potency (and unknown selectivity) of this compound. The authors considered 20 μM a ‘saturating concentration’. Yet in patch‐clamp studies with recombinant Ca_v_1.3‐channels, the concentrations required for 50% inhibition of Ca_v_1.3‐currents was 24 μM, and could be decreased to about 10 μM only at very positive holding potentials (Cooper et al., 2020). Again, these experiments allowed no conclusions regarding the drug's Ca_v_1.3‐selectivity.

Sanchez‐Padilla and colleagues (Sanchez‐Padilla et al., 2014) studied mitochondrial oxidant stress in locus coeruleus neurons in ex vivo mouse brain slices using electrophysiological and optical techniques. Similar to previous findings in SN DA neurons they found that autonomous activity produced phase‐locked dendritic intracellular Ca^2+^‐oscillations. These Ca^2+^‐transients elevated mitochondrial oxidant stress and were due to the activation of LTCCs. To test for a role of Ca_v_1.3, a single concentration of cp‐PYT (50 μM) was tested on both dendritic Ca^2+^‐spikes (observed in the presence of tetrodotoxin) and associated Ca^2+^‐transients. This concentration of cp‐PYT attenuated the amplitude and frequency of Ca^2+^‐spikes and of intracellular Ca^2+^‐oscillations by about 50%. The residual activity was eliminated by isradipine, believed to block additional Ca_v_1.2 channels, which are even expressed at higher abundance than Ca_v_1.3 in these cells (Sanchez‐Padilla et al., 2014). Interestingly, although amplitudes were decreased by cp‐PYT, spikes and transients became much wider, reminiscent of the data from one study (Ortner et al., 2014) showing that the compound can slow activation and inactivation kinetics of both Ca_v_1.3 and Ca_v_1.2 channels, which may have both been modulated by the compound in these cells.

Two publications (Bhandage et al., 2020; Kanatani et al., 2017) found that Toxoplasma gondii‐infection triggers a GABA_A_ ‐dependent hypermigratory phenotype in dendritic cells that requires Ca_v_1.3 Ca^2+^‐signalling. Ca_v_1.3 was the most abundant Ca^2+^‐channel *α*1‐subunit expressed in these cells and further up‐regulated upon infection. The DHPs nifedipine and benidipine (10 μM) abolished this hypermigratory response, an effect seen with an even lower concentration (1 μM) of cp‐PYT. While inhibition of Ca_v_1.3 may explain this effect, Ca_v_1.2 expression in these cells is absent or low (Bhandage et al., 2020; Kanatani et al., 2017), again not permitting any conclusions about selectivity.

Rey et al. (2020) employed cp‐PYT to investigate the role of LTCCs in regulating GABA release from cerebellar molecular layer interneurons (MLIs) in rats. LTCC activation by BayK8644 increased peak Ca^2+^‐currents in MLIs, whereas nimodipine and cp‐PYT had inhibitory effects. cp‐PYT also inhibited action potential‐evoked presynaptic Ca^2+^‐transients and mIPSC frequency in MLIs and Purkinje cells, implicating a presynaptic function of LTCCs in the control of GABA‐release from MLIs. The authors acknowledged published evidence regarding the uncertainty about cp‐PYT selectivity and therefore implicated Ca_v_1.2 or Ca_v_1.3 or a combination of both as responsible for the observed effects.

Given the wide‐spread use of cp‐PYT as a tool compound, it would be of upmost importance to gain a better understanding of its pharmacological profile, not only regarding selectivity for Ca_v_1.3 over Ca_v_1.2, but also with respect to other potential targets.

### 1.7 Sclareol as a natural neuroprotective Ca_v_1.3‐antagonist

A very recent study proposed sclareol (Figure 2a), a constituent of the Mediterranean herb *Salvia sclarea*, as a bioactive compound that inhibits Ca_v_1.3 more strongly than Ca_v_1.2 (Wang et al., 2022). It was discovered in an elegant new cell‐based high‐throughput screening assay. Ca_v_1.2 and Ca_v_1.3 channel complexes were expressed in HEK‐293 cells together with reporter proteins expressed under a synthetic, NFAT3‐based Ca^2+^‐sensitive promotor which responds to K^+^‐depolarization‐induced Ca^2+^ entry through these LTCCs. Surprisingly, screening of 42 essential oil products identified five oils that inhibited Ca_v_1.3 activity with minimal effects on Ca_v_1.2. Using in silico virtual screening and deep learning, 13 hits were identified as the most promising Ca_v_1.3 inhibitors, including (6)‐gingerol and sclareol. However, in their assay no relevant selectivity for these compounds (about 2‐fold, at low micromolar IC_50_ values for both substances, Wang et al., 2022) was found. Although ‘sclareol inhibits Ca_v_1.3 more strongly than Ca_v_1.2’ this small difference cannot be regarded as Ca_v_1.3‐selective. Even though functional Ca_v_1.2 and Ca_v_1.3 channel complexes were expressed in their HEK‐293 cell screening assay, no data from patch‐clamp studies were presented to validate the pharmacological properties of this compound on L‐type Ca^2+^ currents.

Due to earlier findings (see above) suggesting a neuroprotective effect of Ca_v_1.3 inhibition in early PD (see Liss & Striessnig, 2019; Surmeier, Halliday, & Simuni, 2017, for reviews) the authors tested whether this compound has neuroprotective properties in a mouse 6‐hydroxydopamine (6‐OHDA) PD model. Indeed, daily i.p. injections of this compound in mice 2 days before a single unilateral 6‐OHDA injection above the substantia nigra (and not as in most other studies into the striatum) largely prevented the about 50% reduction of staining for the DA neuron‐specific marker tyrosine‐hydroxylase in the ipsilateral dorsal striatum (Wang et al., 2022). This represents indirect evidence for intact SN DA neuron innervation of the striatum and indicates a neuroprotective effect of the drug. A direct, Ca_v_1.3‐dependent neuroprotective effect, evident as the prevention of the loss of vulnerable SN DA neurons, has not been assessed. However, evidence for a protective effect was obtained in behavioural experiments in which locomotor impairments induced by the unilateral 6‐OHDA injections in the vehicle‐treated group (rotational behaviour, hyperlocomotion) were essentially absent in the sclareol‐treated animals. Nevertheless, these findings cannot be taken as a proof for a contribution of Ca_v_1.3 in neuroprotection or in vivo inhibition of Ca_v_1.3. In ex vivo patch‐clamp recordings of putative SN DA neurons in midbrain slices, 10 μM of sclareol caused a large neuronal hyperpolarization (by about 18 mV) accompanied by an increased spiking threshold upon current injection. Such hyperpolarization has not been reported by inhibiting LTCCs with isradipine or in Ca_v_1.3‐deficient SN DA neurons (Guzman et al., 2009; Puopolo et al., 2007). The experiments do not exclude the possibility that the effects of this compound are mediated by other signalling pathways affecting SN DA excitability and do not provide evidence for in vivo activity on Ca_v_1.3.

### 1.8 Ca_v_1.3‐selective toxins

At present no natural compounds, including animal toxins, with Ca_v_1.3‐selectivity have been described. Calciseptine and the structurally highly related FS2 (from black mamba venom) and calcicludin (from green mamba venom) are known LTCC blockers (Wang et al., 2007; Zamponi et al., 2015). In functional studies in Langendorf‐perfused hearts, Barrere et al. (2020) have recently obtained evidence for Ca_v_1.2‐selectivity of calciseptine.

## 2 CONCLUSIONS

Efforts have been made to build on DHP scaffolds to discover new drugs acting more potently on different cardiac functions or in neurons as compared to vascular smooth muscle. Although a number of interesting compounds have been characterized none of them were rigorously tested for Ca_v_1.3‐selectivity. We propose that such selectivity testing should include heterologous systems and/or isolation of native Ca_v_1.3‐mediated (sinoatrial node, inner hair cells and mouse chromaffin cells) or Ca_v_1.2‐mediated (vascular smooth muscle, cardiomyocytes) current components. The concept of defining the selectivity of compounds for Ca_v_1.2 or Ca_v_1.3 channels by performing functional and binding studies on atrial, ventricular and arterial smooth muscle, rat brain cortex and SH‐SY5Y cells without providing evidence for a direct engagement of Ca_v_1.3 channels (such as by demonstrating L‐type current inhibition) is misleading in the important drug discovery process towards potent and highly selective Ca_v_1.3 inhibitors. Cp‐PYT is so far the only compound with some evidence for Ca_v_1.3‐selectivity. However, it is at present unclear under which experimental conditions this selectivity can be observed and solid data obtained in independent laboratories reached different conclusions regarding its potency and selectivity. Overinterpretation of existing data and uncritical reporting cause a substantial overestimation of the suitability of this compound as a Ca_v_1.3‐selective pharmacological tool. Until its activity on other major ionic currents (especially Ca_v_2 and Ca_v_3 Ca^2+^‐channels) has been systematically explored, it should not be used as a pharmacological tool to confirm or refute a role of Ca_v_1.3 channels in cellular responses. This also holds true for sclareol. We therefore conclude that at present there is no published evidence for a validated Ca_v_1.3‐selective compound.

Despite the clear therapeutic rationale and the need for a selective tool for pharmacological studies, it thus appears that the discovery of a compound with selectivity for Ca_v_1.3 over Ca_v_1.2 still remains a considerable challenge.

### 2.1 Nomenclature of targets and ligands

Key protein targets and ligands in this article are hyperlinked to corresponding entries in the IUPHAR/BPS Guide to PHARMACOLOGY http://www.guidetopharmacology.org and are permanently archived in the Concise Guide to PHARMACOLOGY 2021/22 (Alexander, Christopoulos et al., 2021; Alexander, Mathie, et al., 2021).

## AUTHOR CONTRIBUTION

All authors wrote the manuscript and reviewed the final version; LF and TK performed molecular modelling for the illustrations used in this review.

## CONFLICT OF INTEREST STATEMENT

The authors declare no conflict of interest.

## Data Availability

Data sharing is not applicable to this article because no new data were created or analysed in this study. Data for the molecular models used for illustration are available from the corresponding author upon request.
